# Trends in leprosy case detection in Rwanda, 1995–2011: analysis of 17 years of laboratory data

**DOI:** 10.4102/ajlm.v6i1.426

**Published:** 2017-02-28

**Authors:** Innocent Uwimana, Nestor Bizimungu, Fabrice Ingabire, Elyse Mukamukwiye, Odette Sharangabo, Semuto C. Ngabonziza, Elaine Kamanzi

**Affiliations:** 1 School of Public Health, National University of Rwanda, Field Epidemiology and Laboratory Program (FELTP), Kigali, Rwanda.; 2 Rwanda Biomedical Centre/Biomedical Services, National Reference Laboratory Division, Kigali, Rwanda.

## Abstract

**Background:**

Leprosy, or Hansen’s disease, is a chronic, infectious disease caused by *Mycobacterium leprae*. It remains one of the leading causes of deformity and physical disability.

**Objective:**

We analysed laboratory records to assess trends in prevalence rates and case detection rates (CDRs) in Rwanda.

**Methods:**

A retrospective review of detected leprosy cases from the records of the Rwanda National Reference Laboratory over a 17-year period (1995–2011) was conducted. Skin biopsy samples were analysed microscopically using Ziehl-Neelsen staining technique to identify *M. leprae.*

**Results:**

Cumulatively, 266 suspected cases were reported between 1995 and 2011. Of the suspected cases, 77 (28.9%) were laboratory confirmed as having leprosy. Among detected cases, 59 (76.6%) were men and 18 (23.4%) women. The male:female ratio was 3:1. There were 77 registered leprosy cases over the 17-year period of the study, and the prevalence rate was 0.005 per 10 000 population. A gradual decrease in the prevalence rate was observed from 0.015 per 10 000 population in 2003 to 0.003 per 10 000 population in 2010. From 1995 to 2011, the CDR did not exceed one per 10 000 population.

**Conclusion:**

This laboratory review demonstrates a declining trend in prevalence rates and CDR during the period of the study. Early case detection and a sustainable leprosy control programme remain the cornerstones of reducing the physical and socio-economic burden of leprosy in Rwanda.

## Introduction

Despite the availability of powerful, multi-drug therapies, leprosy remains one of the world’s most infectious diseases and is a leading cause of deformity and physical disability. Leprosy, or Hansen’s disease, is a chronic infection caused by *Mycobacterium leprae*. For affected patients, leprosy carries a significant stigma and contributes to their isolation from the rest of the world. The global burden of leprosy in 1993 was estimated at 2.4 million of leprosy cases worldwide against 10 to 12 million in 1980, and 5.4 million in 1985.^1^ In 2010, the registered prevalence of leprosy worldwide was 211 903 cases, with the World Health Organization (WHO) reporting 244 796 new cases detected during 2009.^2^ The WHO reported that the highest number of cases occurred in South East Asia (*n* = 166 115 new cases), followed by the African Region (*n* = 28 934 new cases).^2^

In 1991, the WHO issued a resolution to achieve global elimination of leprosy by 2000, with ‘elimination’ defined as a reduction in prevalence of the disease to less than 1 case per 10 000 population.^3^ Although this target was achieved in 2000, many countries continue to experience transmission of the disease. Currently, leprosy remains endemic in several countries, including India, Indonesia and Brazil, which have the highest global burden of leprosy worldwide.^4^ In the WHO Americas region, Brazil ranks first with 19.2%, followed by Suriname with 7.2% and Paraguay with 6.3%. In the WHO’s Eastern Mediterranean Region, Sudan had 4.9% and Qatar 2.8%. In the Southeast Asia Region, Nepal detected 14.8%, East Timor 14.5% and India 11.0%. In the Western Pacific Region, Kiribati had 95.3%, Marshall Island had a detection rate of 80.8% and Nauru 30.0%. In the WHO African Region, Comoros represented the highest detection rate, with 44.3% cases per 100 000 inhabitants in 2009, followed by Liberia with 10.8% and Sierra Leone with 8.0%.^5^

The WHO’s introduction of multi-drug therapy, a combination of three drugs – dapsone, rifampicin and clofazimine – has contributed spectacularly to the improvement of leprosy case management and disease control. Use of this multi-drug therapy in association with political commitment has decreased the prevalence of leprosy worldwide in several countries, strengthening disease surveillance by coordinating available internal and external resources for leprosy control and striving for integration of leprosy control into general health services for an inclusive and better disease surveillance.^6,7^ In 1995, the Rwanda National Tuberculosis and Leprosy Control Programme initiated efforts to increase detection and treatment of leprosy cases by engagement of community health workers and education for awareness of leprosy to the community. Despite strong measures put in place to eliminate leprosy, Rwanda is still recording new cases. In 2010, eight new cases with grade-2 disabilities were recorded in Rwanda. The present study aimed to assess the trends in the prevalence rates and case detection rates (CDRs) in Rwanda to evaluate whether the country meets the WHO leprosy elimination target.

## Methods

### Ethical considerations

This study was conducted after obtaining authorisation from the Ethical Committee of the School of Public Health, University of Rwanda. The authorisation for using laboratory records was sought from the Rwanda Biomedical Centre, National Reference Laboratory Division. No authorisation numbers were issued. No patients’ names were used during data collection or analysis, as patients were delinked from their names and codes were used during data collection.

### Setting, study period and study population

This retrospective analysis of leprosy cases detected over a 17-year period was conducted using data from the laboratory records of the Rwanda National Reference Laboratory Division in Kigali, Rwanda. The National Reference Laboratory is a referral laboratory dedicated to performing specialised laboratory tests from various health facilities, including intermediate district hospitals and health centres at the peripheral level. The National Reference Laboratory receives and performs laboratory tests for all suspected cases of leprosy countrywide. Data were retrieved from laboratory registers for smear microscopy examinations performed from January 1995 to December 2011. Examinations were conducted for all suspected cases of leprosy on samples collected from patients who attended any health facility in Rwanda.

### Laboratory tests and interpretation

Exudates from slit skin were collected and sent immediately to the National Reference Laboratory Division. Smears were stained by using the hot Ziehl-Neelsen staining technique to detect acid alcohol-resistant bacilli in skin smears collected from skin lesions, ear lobes, elbows and/or other exudates. The Ziehl-Neelsen test was used to assess bacillus morphology and the bacterial index. The bacterial index represents the quantitative bacillary load (number of bacilli) and is expressed according to a logarithmic scale ranging from 0 to 4+. A positive smear was classified as ‘multibacillary’ if its bacterial index was evaluated to be 1+ to 4+ and as ‘paucibacillary’ if its bacterial index was either negative or scanty.^8,9^ Leprosy smear microscopy interpretation was done by a well-trained laboratory technologist to identify *M. leprae*. The WHO recommends definitive identification and confirmation of leprosy if one of the two criteria are found: (1) skin biopsy smear positive; or (2) characteristic anaesthetic leprosy skin lesions, with or without nerve thickening or enlargement, with sensory or motor loss.^10^

### Data collection and analysis

Data collected from the laboratory registers were entered in Epi-Info (version 3.5.3). Distribution of variables, including age, sex and quantitative bacillary load, was collected and analysed by describing simple frequencies. The case detection rate (CDR), an important indicator being used under the National Leprosy Eradication Programme, and prevalence rate per 10 000 inhabitants were calculated according to WHO guidelines, as described elsewhere. Briefly, CDR was calculated based on the number of cases detected in a year multiplied by 100 000 and divided by the total population in that year.^11,12,13^

## Results

**FIGURE 1:**
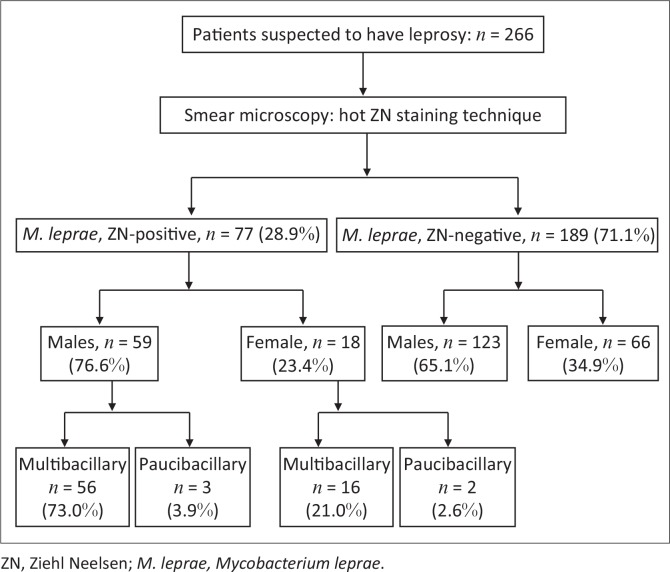
Results of enrolled patients for laboratory detection of *Mycobacterium leprae*, Rwanda, 1995–2011.

A total of 266 suspected cases were reported between 1995 and 2011 (Figure 1). Of the suspected cases, 77 (28.9%) were laboratory confirmed as being infected with *M. leprae.* The remaining cases (*n* = 189; 71.1%) were microscopically negative for acid alcohol-resistant bacilli or leprosy bacilli. Among the detected cases, 76.6% (*n* = 59) were men and 23.4% (*n* = 18) women. The male:female ratio was 3:1. The most affected age group was the group of patients over age 45 years. Multibacillary patients with a bacteriological index ranging from 1+ to 4+ were detected in 73.0% of men and 21.0% of women. Paucibacillary cases were less common, at 3.9% in men and 2.6% in women (Figure 1).

**FIGURE 2:**
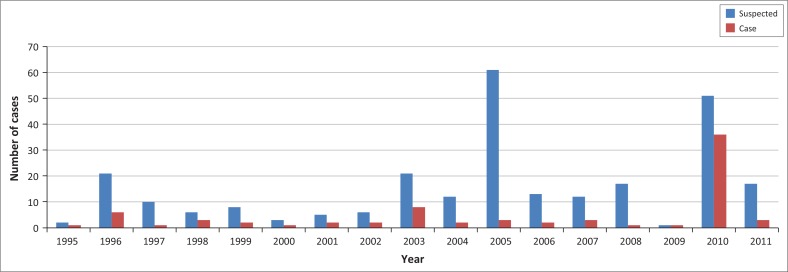
Suspected cases compared to laboratory-confirmed cases, Rwanda, 1995–2011.

The highest number of cases was recorded in 2010, with a CDR of 0.33 per 100 000 population. In 2005, more suspected cases (23.0%; 61/266) were recorded, but the CDR was lower (0.03 per 100 000 inhabitants) (Figure 2).

**FIGURE 3:**
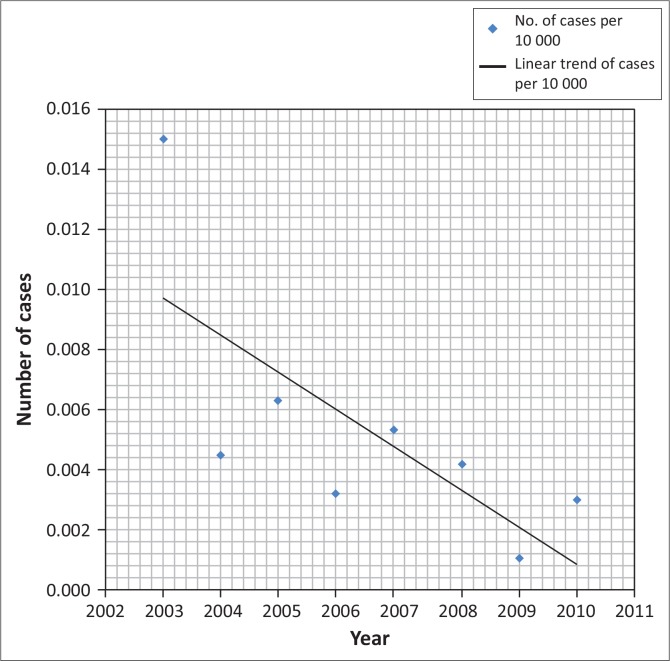
Prevalence rate of leprosy in Rwanda from 2003–2010.

There were 77 registered leprosy cases over the 17-year study period, with a prevalence rate of 0.005 per 10 000 population. The prevalence rate decreased from 0.015 per 10 000 population in 2003 to 0.003 per 10 000 population in 2010 (Figure 3). From 1995 to 2011, the CDR did not exceed one per 10 000 inhabitants.

## Discussion

The present study shows that over a period of 17 years, the prevalence of leprosy in Rwanda has decreased and remained below the WHO’s elimination target of less than 1 case per 10 000 population. This might be due to increased awareness amongst community health workers, as well as a community sensitisation effort for case detection and treatment.

Annually, the WHO describes and provides regional CDRs from individual national CDRs in a listing of the top 33 endemic countries and top 14 individual countries. In 1998, the WHO found that in seven of the top 14 countries, including India and Brazil, the CDR was above two per 10 000. India contributed 79% to global case detection in the same year. The African, American and Southeast Asian WHO Regions each accounted for about 30%, once India was excluded. Between 1994 and 2000, case detection did not decrease in these three WHO regions, according to the same estimates.^14^ Our study shows that Rwanda can be classified among the countries that have achieved the WHO target phase of leprosy elimination (prevalence rate < 1/10 000).

The gradual decrease in leprosy prevalence rates shows that Rwanda has reached the WHO target of leprosy elimination as it was defined in 1991.^15^ Studies conducted elsewhere, such as in Togo, Lome, where a similar retrospective analysis was conducted from January 1990 to December 2005, found similar decreases in leprosy prevalence trends, with prevalence and CDR dropping remarkably from 1990 to 2005.^16^ In our analysis, leprosy case detection and the prevalence rates dropped noticeably over an eight-year period, from 0.015 per 10 000 population in 2003 to 0.003 per 10 000 population in 2010, and from 1995 to 2011 the CDR did not exceed one per 10 000 inhabitants. This gradual decrease was the result of the good surveillance system established countrywide.

Although the CDR in Rwanda did not exceed one per 10 000 inhabitants from 1995 to 2011, multibacillary patients with a bacteriological index ranging from 1+ to 4+ were highly represented in our analysis. Similar CDRs are found elsewhere in the world. An epidemiological survey on leprosy in metropolitan France found a CDR of 0.003 per 10 000 inhabitants.^17^ In French Guyana, near the border with Brazil, a study found a CDR of 0.53 cases/10 000 inhabitants/year; this area had the highest number of leprosy cases in the world and ranked second worldwide after India, which in 2009 detected the highest number of new cases.^1,18^ The low detection of leprosy cases among women in our study lead to a 3:1 male:female ratio, which could be explained by better adherence to drug regimens among women when compared to men, as has been found elsewhere.^19^

### Limitations

There are two major limitations for the present study. First, we were not able to trace and study patient treatment outcomes. Additionally, because this was a retrospective data analysis from routine laboratory records, some information was missing, which hampered our ability to analyse some important variables.

### Recommendations

We recommend early case detection through an active case detection strategy for a sustainable leprosy control programme. Further studies and nationwide surveys are recommended for continuous monitoring of leprosy, patient treatment outcomes and its estimation for better disease surveillance and case management countrywide.

### Conclusion

Our study shows that Rwanda has achieved the WHO global leprosy elimination target of less than one case per 10 000 and has demonstrated a declining trend in leprosy prevalence and CDRs. The attainment of the WHO leprosy elimination target became a reality in Rwanda by putting various strategies and interventions in place, including sensitisation of community health workers, active disease surveillance using active samples and data collection, and political will. These strategies are the cornerstone that will allow reduction of physical disabilities and socio-economic burden of leprosy in the country.
